# Tension hemothorax due to iatrogenic subclavian artery perforation: Hybrid management of a very rare complication

**DOI:** 10.1016/j.ijscr.2019.06.024

**Published:** 2019-06-20

**Authors:** Stefano Magnone, Riccardo Gotti, Michela Giulii Capponi, Nadiane Paderno, Cosimo Maraglino, Manuela Cadei, Consuelo Mario, Alessandro Lucianetti

**Affiliations:** a First General Surgery Unit, Bergamo, Italy; b Vascular Surgery Unit, Bergamo, Italy; c Fist Anesthesiology Service, Bergamo, Italy

**Keywords:** Hemothorax, Obstructive shock, Hemorrhagic shock, Subclavian artery

## Abstract

**Background:**

Tension hemothorax is a rare event, due to different causes: trauma, ruptured thoracic aorta aneurysms, or as a complication of central venous line placement due to inadvertent artery puncture or cannulation. Tension hemothorax leads to both hypovolemic and obstructive shock and can require emergency management.

**Presentation of case:**

A 63 years old lady underwent a complicated surgical procedure for a postoperative small bowel obstruction after radical cystectomy. During the procedure, a central venous catheter was placed, under ultrasound guidance, in the right jugular vein but an unknown puncture of the right subclavian artery occurred. In the early phase of the postoperative course, a hypovolemic/obstructive shock developed because of a tension hemothorax. The patient underwent an emergency thoracotomy in the hybrid room, followed by an endovascular repair of the arterial laceration. A recurrent hemothorax developed a few hours later because of an endoleak that was treated successfully with a second endovascular approach and a balloon dilatation of the stent.

**Conclusions:**

tension hemothorax due to inadvertent subclavian artery laceration can be life-threatening and should be managed in a hybrid room with endovascular and surgical capabilities.

## Highlights

• Tension hemothorax is a life threatening condition.
• Emergency thoracotomy can be accomplished in a hybrid room.
• Endovascular subclavian artery stenting is a good choice in an emergency setting.

## 1 Introduction

Tension hemothorax is a rare event, due to different causes: trauma with fracture of the first rib [1,2], ruptured thoracic aorta aneurysms [3,4], or as a complication of central venous line placement due to inadvertent artery puncture or cannulation [5]. Tension hemothorax leads to both hypovolemic and obstructive shock and request emergency management. Our case report is in line with the SCARE criteria [6].

## 2 Case presentation

**Fig. 1:**
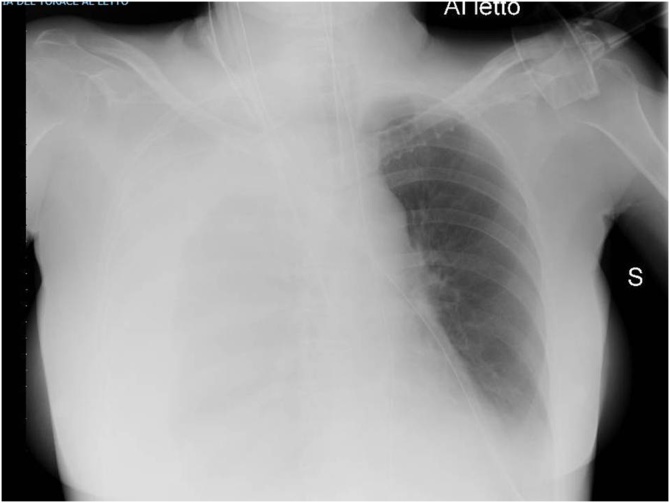
Hemothorax on chest X-ray.

**Fig. 2:**
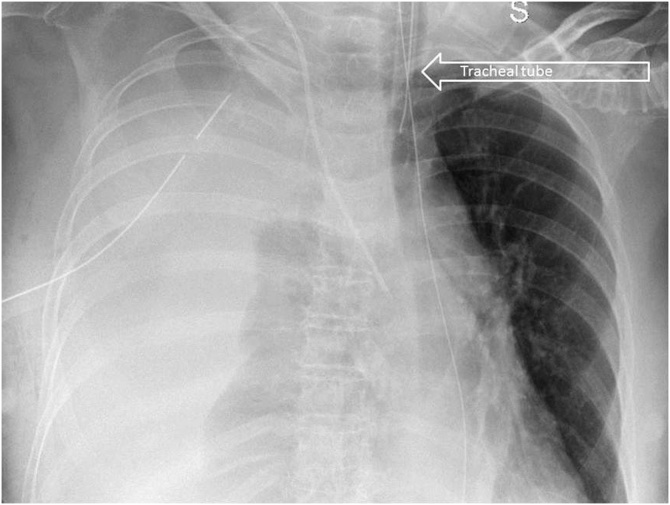
Right tension hemothorax. See the tracheal deviation toward the left.

**Fig. 3:**
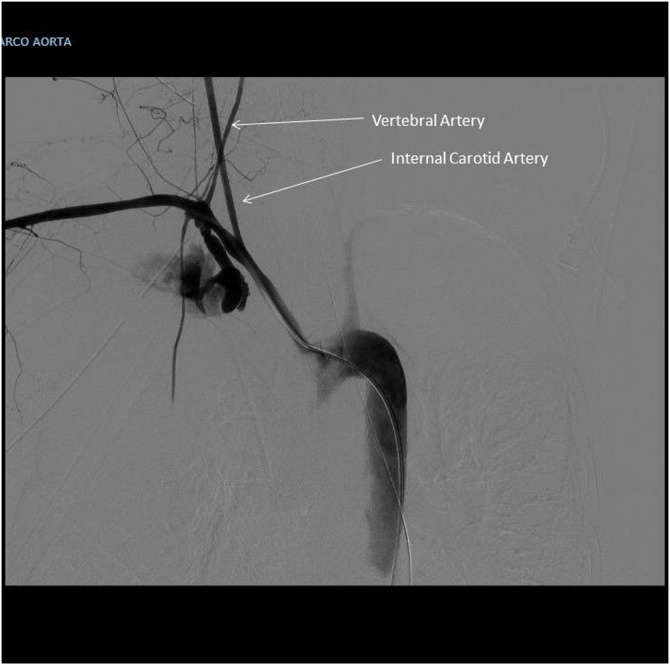
Massive bleeding from the right subclavian artery on angiography.

**Fig. 4:**
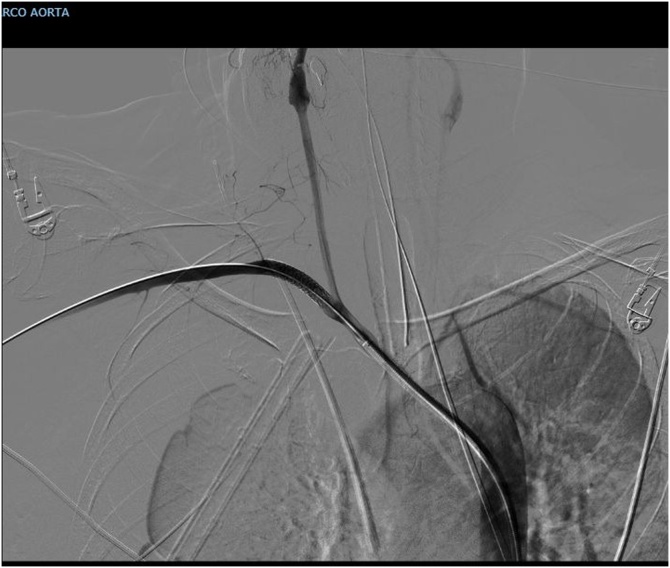
Balloon dilatation to deploy the stent.

**Fig. 5:**
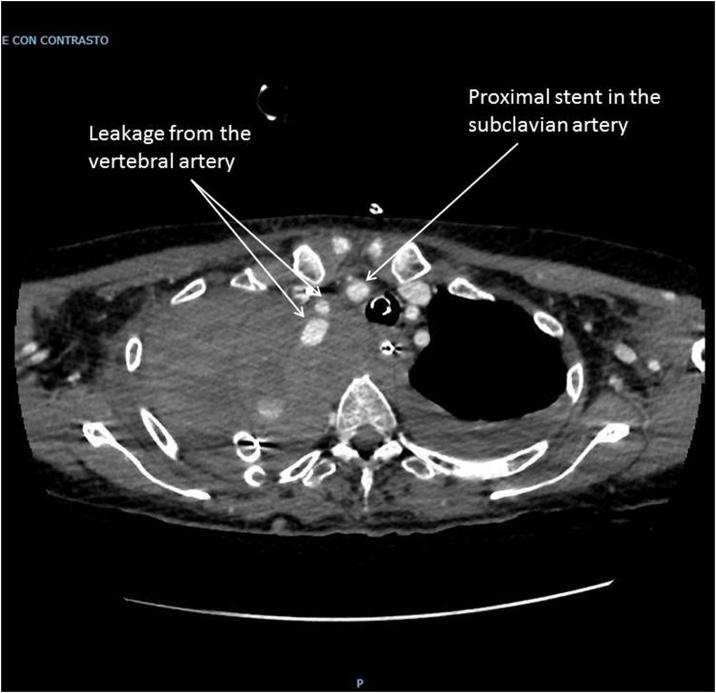
CT scan showing leakage from the stent, due to reperfusion of the vertebral artery a few hours after the angiographic procedure.

**Fig. 6:**
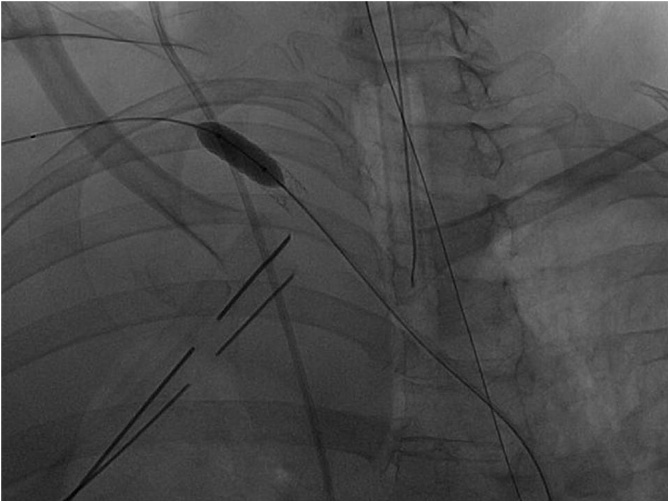
Second balloon dilatation to control retrograde bleeding from the vertebral artery.

A 63 years old lady carried out a complex re-laparotomy for a postoperative small bowel occlusion after a radical cystectomy because of urothelial carcinoma. The procedure lasted 3 h because of thick adhesions that needed to be cleared. At the end of the procedure, total blood loss was 2 litres, and two Units of Packed Red Blood Cells (PRBC) were infused. During the surgical procedure, a central venous catheter was placed in the internal jugular vein by ultrasound-guided puncture of the vessel, but an inadvertent puncture and cannulation of the right subclavian artery occurred before catheter placement. Because of the ultrasound-guided procedure, the anesthesiologist thought to have cannulated the carotid artery and applied local pressure for a few minutes. Two hours later, when the patient was in the Intensive Care Unit (ICU) around midnight, a chest X-ray to check the correct position of the central venous catheter revealed a massive hemothorax (Fig. 1), while the patient was hypotensive and responder to crystalloids and blood infusions. A chest drain was inserted without any substantial output but a small amount of clotted blood. The patient rapidly worsened, despite appropriate resuscitation with 10 U of PRBC, 8 U of Fresh Frozen Plasma (FFP), 3 U of cryoprecipitate and 1 U of platelets from apheresis. A second chest X-Ray to check tube position revealed a tension hemothorax (Fig. 2). The team in charge of the patient, comprising the anesthesiologist that did the general anaesthesia for the surgical procedure, made the diagnosis of suspected subclavian artery perforation and tension hemothorax with both hypovolemic and obstructive shock. The team decided to bring the patient in the hybrid room to control the likely bleeding for the right subclavian artery. While the patient has been positioning on the angiographic table, the ECG monitor showed severe bradycardia, with a heart rate of 30 and an impending cardiac arrest with a systolic blood pressure of 30 mmHg. An emergency thoracotomy to decompress the right chest was then performed via a V space incision, and five litres of blood under pressure were drained from the right pleural space, with a rapid improvement in the vital signs. During the endovascular procedure, the general surgeon left the thoracotomy open to allow a continuing suction in the pleural space to prevent the accumulation of clots. The endovascular procedure was conducted by the vascular surgeons and lasted 40 min, confirming the massive leak from a significant defect in the subclavian artery (Fig. 3) and consisted in a 7 x 37 mm covered stent placement in the subclavian artery at the origin of the vertebral artery. The stent was expanded with a balloon taking care of not injuring the already damaged vessel (Fig.4). The patient had a transient improvement, but in the next few hours, a hemodynamic instability again occurred, even if responsive to blood infusion. A CT scan revealed a leakage from the stent, because of retrograde revascularization of the vertebral artery (Fig. 5). A new endovascular procedure, with more pronounced balloon dilatation of the stent, definitely controlled the bleeding. Fig.6) The patient slightly improved after an open surgical debridement of the pleural space from clots and blood, given the absence of an output from the two large drains that were in place. In the next few days, the patient went back to the ward and made a gradual full recovery with no neurological or vascular defects (Table 1).

**Table 1:** Dates Relevant Past Medical History and Interventions.

May 10, 2018	Radical cystectomy for urothelial carcinoma. Subsequent development of acute small bowel obstruction (ASBO)		
Date	Summaries from Initial and Follow-up Visits	Diagnostic Testing (including dates)	Interventions
June 2, 2018, 08.00 pm	ASBO requiring laparotomy.	CT scan	Midline re-laparotomy. Extensive adhesiolysis.
June 3, 2018 01.00 am	Massive hemothorax	Chest Xray	Chest Drain
June 3, 2018 04.00 am	Hemorrhagic shock and tension hemothorax.	Chest Xray	Emergency thoracotomy. Angiography and subclavian artery stenting.
June 3, 2018, 08.00	Re-bleeding from the vertebral artery	CT scan	Angiography, re-ballooning of the stent
Mid-July	Discharge. Short bowel syndrome.		

## 3 Discussion

Tension hemothorax is a well-known complication of trauma and thoracic aortic aneurysm rupture [[1], [2], [3], [4]], while central venous line placement usually causes massive hemothorax [5]. In this case the complication of central venous catheter placement caused a subclavian artery laceration and a pleural lesion that led to a far rarer complication such as tension hemothorax, which is the cause of a hypovolemic and obstructive shock at the same time. In this skinny lady, the ultrasound approach did not protect the manoeuvre, done by a very skilled anesthesiologist. The hybrid room permitted a prompt surgical and endovascular treatment of this life-threatening condition, as in others’ experience [7]. In this case a Video-Assisted-Thoracoscopy (VATS) was not considered a valid option because of the emergency, with an impending cardiac arrest in place, and the need for a rapid procedure whose purpose was just the chest decompression. A recurrent leak could be prevented with more pronounced balloon dilatation of the stent at the time of placement; this was not accomplished at that time because of fear for artery complete disruption.

## 4 Conclusions

Tension hemothorax due to inadvertent subclavian artery laceration is an infrequent complication of a standard procedure, like central venous line placement. It can be life-threatening and should be managed in a hybrid room with endovascular and surgical capabilities. We presented a case which requested an emergency thoracotomy to resolve an obstructive shock before the endovascular stenting of the arterial laceration.

## Conflicts of interest

Authors declare no conflicts of interest.

## Funding

No funding.

## Ethical approval

This is a case report, IRB approval is not requested.

## Consent

We obtained patient’s consensus (written and signed) for publication.

## Author’s contribution

SM, AL. Substantial contribution to the study conception and design, data acquisition, analysis, and interpretation.

SM. Drafting or revising the article for intellectual content.

SM, RG, MGC, NP, CM, MC, CM, AL. Agreement to be accountable for all aspects of the work related to the accuracy or integrity of any part of the work.

SM, RG, MGC, NP, CM, MC, CM, AL. Approval of the final version

## Registration of research studies

N.A.

## Guarantor

Stefano Magnone (first and corresponding author).

## Provenance and peer review

Not commissioned, externally peer-reviewed.
